# Combined Targeting of AKT and mTOR Inhibits Proliferation of Human NF1-Associated Malignant Peripheral Nerve Sheath Tumour Cells In Vitro but not in a Xenograft Mouse Model In Vivo

**DOI:** 10.3390/ijms21041548

**Published:** 2020-02-24

**Authors:** Alexander Schulte, Florian Ewald, Melanie Spyra, Daniel J. Smit, Wei Jiang, Johannes Salamon, Manfred Jücker, Victor-Felix Mautner

**Affiliations:** 1 Laboratory for Tumor Genetics, Department of Neurology, University Medical Center Hamburg-Eppendorf, Martinistraße 52, 20246 Hamburg, Germany; alexan.schulte@gmail.com (A.S.); melaniespyra1@gmx.de (M.S.); w.jiang@uke.de (W.J.); v.mautner@uke.de (V.-F.M.); 2 Department of General, Visceral and Thoracic Surgery, University Medical Center Hamburg-Eppendorf, Martinistraße 52, 20246 Hamburg, Germany; f.ewald@uke.de; 3 Institute of Biochemistry and Signal Transduction, University Medical Center Hamburg-Eppendorf, Martinistraße 52, 20246 Hamburg, Germany; d.smit@uke.de; 4 Department of Diagnostic and Interventional Radiology and Nuclear Medicine, University Medical Center Hamburg-Eppendorf, Martinistraße 52, 20246 Hamburg, Germany; j.salamon@uke.de

**Keywords:** MPNST, targeted therapy, AKT, neurofibromatosis type 1, signaling, xenograft model

## Abstract

Persistent signalling via the PI3K/AKT/mTOR pathway is a major driver of malignancy in NF1-associated malignant peripheral nerve sheath tumours (MPNST). Nevertheless, single targeting of this pathway is not sufficient to inhibit MPNST growth. In this report, we demonstrate that combined treatment with the allosteric pan-AKT inhibitor MK-2206 and the mTORC1/mTORC2 inhibitor AZD8055 has synergistic effects on the viability of MPNST cell lines in comparison to the treatment with each compound alone. However, when treating animals bearing experimental MPNST with the combined AKT/mTOR regime, no influence on tumour growth was observed. Further analysis of the MPNST xenograft tumours resistant to AKT/mTOR treatment revealed a reactivation of both AKT and mTOR in several tumour samples. Additional targeting of the RAS/RAF/MEK/MAPK pathway with the allosteric MEK1/2 inhibitor AZD6244 showed synergistic effects on the viability of MPNST cell lines in vitro in comparison to the dual AKT/mTOR inhibition. In summary, these data indicate that combined treatment with AKT and mTOR inhibitors is effective on MPNST cells in vitro but tumour resistance can occur rapidly in vivo by restoration of AKT/mTOR signalling. Our data further suggest that a triple treatment with inhibitors against AKT, mTORC1/2 and MEK1/2 may be a promising treatment option that should be further analysed in an experimental MPNST mouse model in vivo.

## 1. Introduction

Neurofibromatosis Type 1 (NF1) is an autosomal dominant disease that is caused by loss of function-mutations and/or deletions in the tumour suppressor gene *neurofibromin 1* (*NF1*). NF1 is either inherited or occurs de novo, with a cumulative incidence of 1 case in 3.000 new-born individuals diagnosed worldwide each year [1,2]. Neurofibromin is a negative regulator of the rat sarcoma (RAS) proto-oncogenes, and a loss of function in *NF1* enhances RAS-dependent and subsequent activation of the mitogen-activated protein kinase (MAPK) pathway and the PI3K/AKT/mTOR pathway, which have been demonstrated to be essential for NF1-associated malignancies [3,4].

Up to 90% of NF1 patients develop NF1-associated tumours called neurofibromas. Two out of three neurofibromas, and therefore the vast majority of all neurofibromas, are benign cutaneous tumours, which usually do not develop before puberty and do not transform to malignancy [5]. Around 30% [6] of NF1 patients will have benign plexiform neurofibromas (PNF), which are externally visible and are often located in the face, neck, hip or lower leg [5]. The frequency of PNF increases to 50% when patients are investigated by whole-body MRI, which detects internal tumours [7]. Unlike cutaneous neurofibromas, PNF are already present at birth and can increase in size proportional to the patient’s body weight but do not develop de novo at higher age. Nevertheless, the plexiform lesions in NF1 patients, although present from birth, are not always visible at that point. Most importantly, PNF can progress to malignant peripheral nerve sheath tumours (MPNST) with a lifetime risk of 8–13% [8,9,10,11]. However, little is known about the underlying molecular mechanisms and the risk factors for malignant progression [12]. Although the malignant transformation of PNF is not the most common complication (8–13% lifetime risk) in Neurofibromatosis Type 1 patients, MPNST are associated with the highest mortality among complications, with a 5-year survival rate of less than 30% [8,10,11]. Surgery is mostly palliative in NF1 patients, due to the highly aggressive growth of MPNST, their strong tendency for metastatic spread and the location of the tumours in the close vicinity of vital internal organs [7].

Current treatment options, including radio- and chemotherapy, have shown only little efficacy in MPNST [13,14]. In preclinical models, pharmacological inhibition of the RAS/RAF/MEK/MAPK cascade has been demonstrated to slow down tumour growth and increase overall survival of mice bearing MPNST xenografts [15]. Additionally, targeting the mTOR pathway by rapamycin has also been demonstrated to have an effect on NF1-associated tumours in an engineered mouse model of NF1 [3]. Dual targeting of PI3K/mTOR by PI-103 and mTOR by rapamycin has been proposed as a potential therapeutic strategy for MPNST [16]. However, rapamycin only targets the mTORC1 component of the mTOR multiprotein complex, whereas mTORC2 is essential for the activation of AKT. In 2016, Varin et al. further demonstrated that dual mTORC1/2 inhibition can induce antiproliferative effects in NF1-derived MPNST cell lines in vitro [17]. Additional inhibition of the MEK/ERK MAPK pathway showed synergism in reducing viability of MPNST cell lines. The activity of AKT and mTOR is crucial for the malignant behaviour of NF1-associated neoplasms such as MPNST or optic pathway gliomas [16,18,19], as well as for other tumour entities such as hepatocellular carcinoma and cholangiocarcinoma [20,21,22].

In our recent experiments, we were able to recapitulate the findings from Varin et al. [17] on mTORC1/mTORC2 inhibition in additional NF1-associated MPNST cell lines. Additionally, we demonstrate that dual targeting of AKT with the allosteric pan-AKT inhibitor MK-2206 and mTOR using the mTORC1/mTORC2 bi-specific ATP-competitor AZD8055 is sufficient to significantly decrease NF1-null MPNST cell viability in vitro. However, we find that this combination, in spite of the promising results in vitro, is insufficient to inhibit MPNST growth in a subcutaneous xenograft mouse model in vivo. Furthermore, we show that additional inhibition with the MEK inhibitor AZD6244 shows synergistic effects on the viability of MPNST cells in vitro.

## 2. Results

### 2.1. Inhibition of AKT and mTOR Alone Reduces Cell Viability of MPNST Cells In Vitro

**Figure 1:**
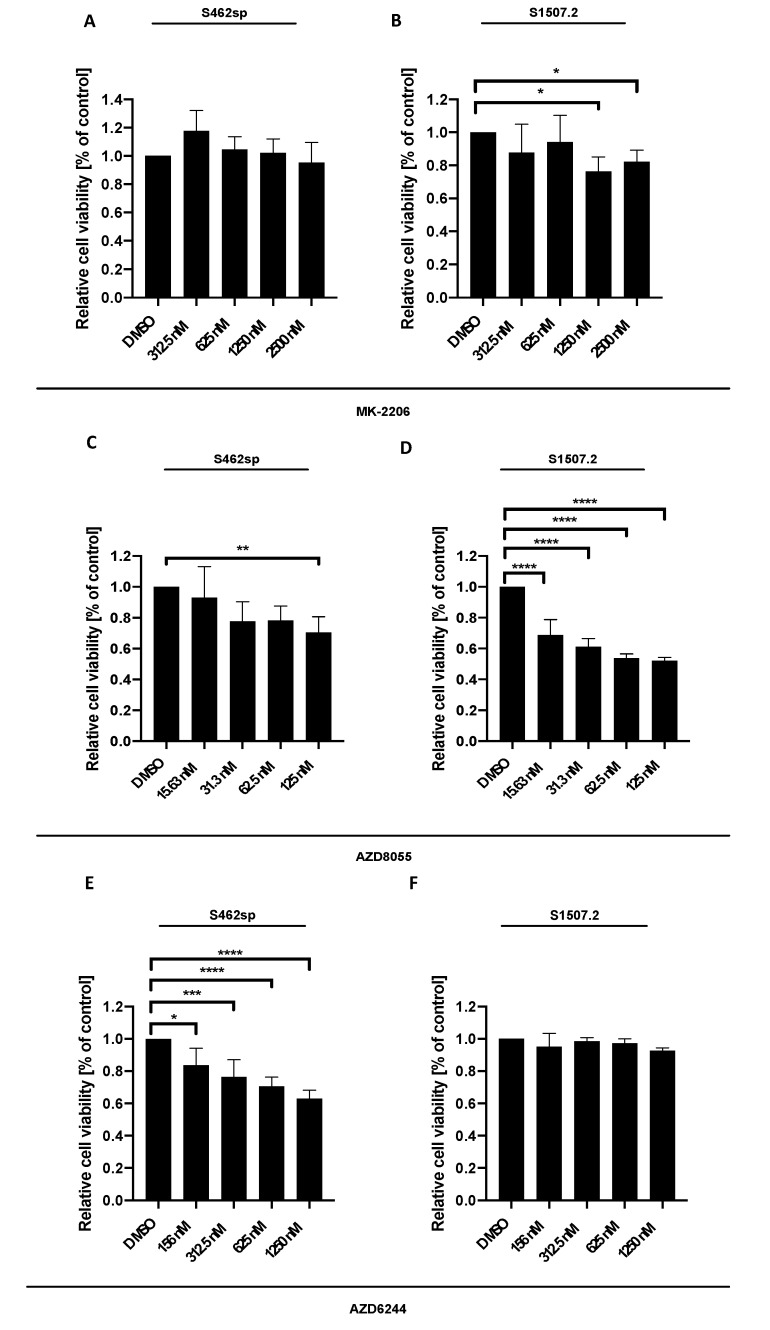
MPNST cell viability can be decreased targeting either AKT, mTORC1/2 or MEK1/2 in vitro. MPNST cells were seeded in a 96-well-plate at a density of 5000 cells per well. Relative cell viability (normalised to control) of S462sp (**A**, **C**, **E**) and S1507.2 (**B**, **D**, **F**) cells under varying concentrations of either MK-2206 (A, B), AZD8055 (C, D) or AZD6244 (E, F) was measured by XTT assay after 72 h. The mean values with standard deviation are shown. Statistical significance was analysed with a one-way ANOVA with Dunnett’s multiple comparisons test and compared to the vehicle substance dimethyl sulfoxide (DMSO) (* *p* ≤ 0.05; ** *p* ≤ 0.01; *** *p* ≤ 0.001; **** *p* ≤ 0.0001).

MPNST cells S462 cells grown as neuropheres (referred to as S462sp) or S1507.2 were treated with different concentrations of either MK-2206 [23], AZD8055 or AZD6244, in vitro, and cytotoxicity was measured by XTT assay after 72 h of incubation. The three inhibitors showed varying significant effects on the viability of the two MPNST cell lines analysed, with some differences regarding the concentration-dependent response (Figure 1).

### 2.2. Combined Inhibition of mTOR and AKT Shows Synergistic Impact on S462sp and S1507.2 Cell Viability In Vitro

**Figure 2:**
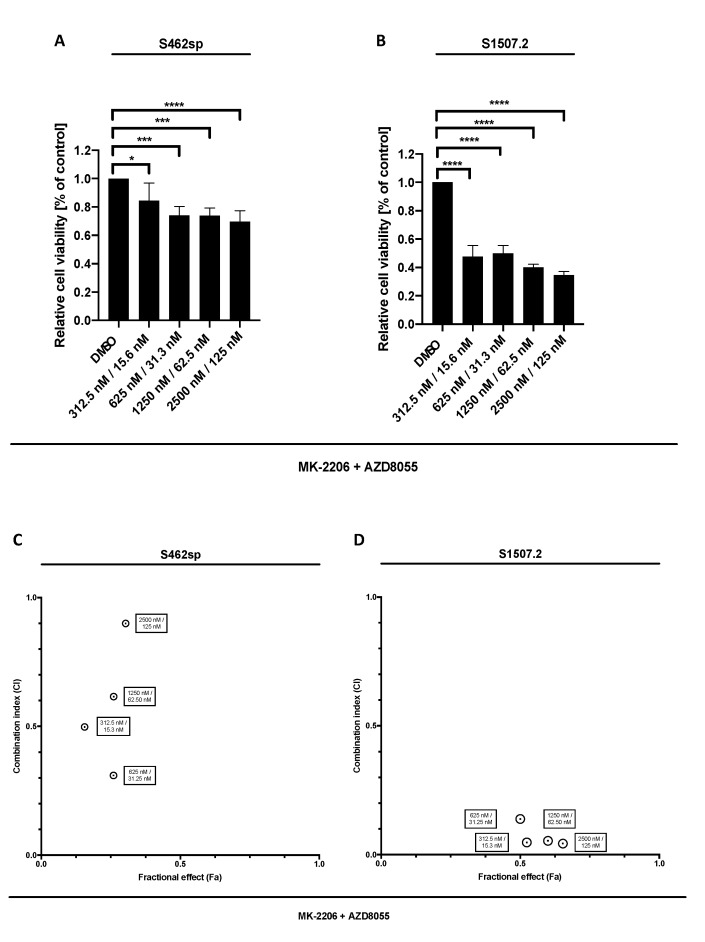
MPNST cell viability after combinatory treatment with MK-2206 and AZD8055 in vitro. MPNST cells were seeded in a 96-well-plate at a density of 5000 cells per well. Relative cell viability (normalised to control) of S462sp (**A**) and S1507.2 (**B**) cells under varying concentrations of combined MK-2206 and AZD8055 treatment was measured in an XTT assay after 72 h. The mean values with standard deviation are shown. Statistical significance was analysed with a one-way ANOVA with Dunnett’s multiple comparisons test and compared to the vehicle substance dimethyl sulfoxide (DMSO) (* *p* ≤ 0.05; *** *p* ≤ 0.001; **** *p* ≤ 0.0001). Combination indices (CI) were calculated based on the XTT assay (Figure 1 and Figure 2) according to the Chou and Talalay method and plotted against their respective fractional effect (Fa) on cell viability (very strong synergism CI < 0.1; strong synergism CI 0.1–0.3; synergism CI 0.3–0.7; moderate synergism CI 0.7–0.85; slight synergism CI 0.85–0.90; nearly additive CI 0.9–1.10; antagonistic CI > 1.1). The data for dual targeting with MK-2206 and AZD8055 on S462sp (**C**) and S1507.2 (**D**), compared to single treatment, are shown.

In a second step, we treated the cell lines with a combination of MK-2206 and AZD8055 in order to determine a possible synergism of dual AKT/mTOR targeting in vitro. Indeed, combined therapy led to a dose-dependent reduction of viability in the two cell lines analysed (Figure 2A,B). Calculation of combination indices according to the Chou and Talalay method [24] revealed synergistic effects in dual targeting of the AKT/mTOR signalling in S462sp in the four concentrations tested (Figure 2C,D) and very strong to strong synergistic effects in all tested concentration of MK-2206/AZD8055 in S1507.2 cells (Figure 2D).

### 2.3. Additional Targeting of MEK1/2 in Combination with Inhibition of AKT and mTOR Further Decreases Viability in MPNST Cell Lines in Vitro 

**Figure 3:**
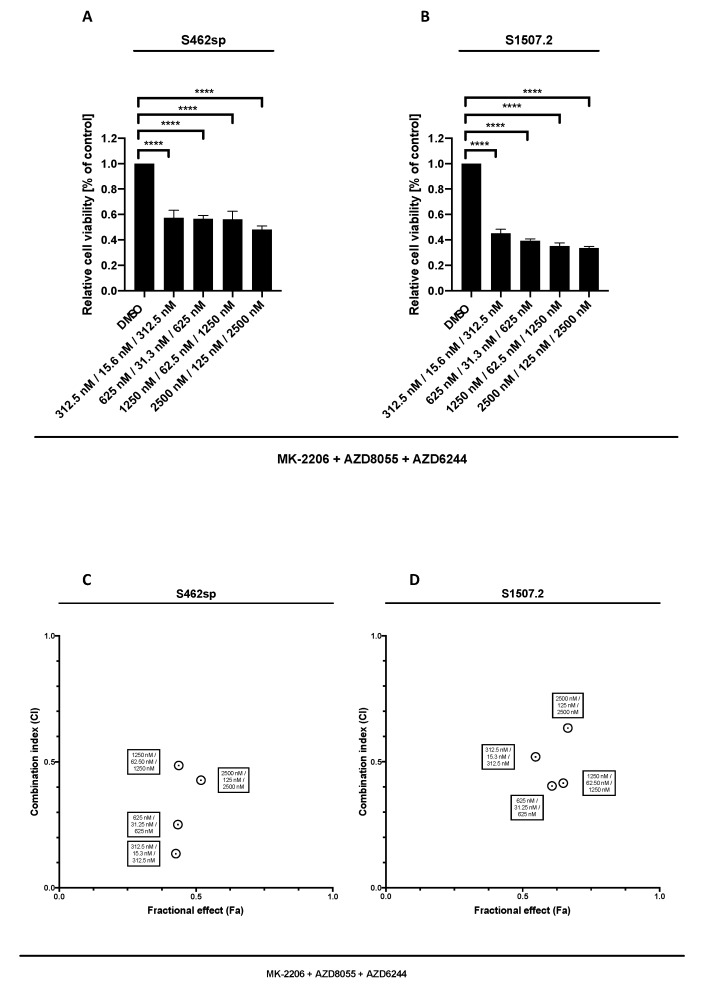
MPNST cell viability after triple treatment with MK-2206, AZD8055 and AZD6244 in vitro. MPNST cells were seeded in a 96-well-plate at a density of 5000 cells per well. Relative cell viability (normalised to control) of S462sp (**A**) and S1507.2 (**B**) cells under varying concentrations of combined MK-2206, AZD8055 and AZD6244 treatment was measured in an XTT assay after 72 h. The mean values with standard deviation are shown. Statistical significance was analysed with a one-way ANOVA with Dunnett’s multiple comparisons test and compared to the vehicle substance dimethyl sulfoxide (DMSO) (**** *p* ≤ 0.0001). Combination indices (CI) were calculated based on the XTT assay (Figure 2 and Figure 3) according to the Chou and Talalay method and plotted against their respective fractional effect (Fa) on cell viability (very strong synergism CI < 0.1; strong synergism CI 0.1–0.3; synergism CI 0.3–0.7; moderate synergism CI 0.7–0.85; slight synergism CI 0.85–0.90; nearly additive CI 0.9–1.10; antagonistic CI > 1.1). The data for triple targeting with MK-2206, AZD8055 and AZD6244 on S462sp (**C**) and S1507.2 (**D**) compared to dual treatment with MK-2206 and AZD8055 is shown.

Our results show that targeting MEK1/2 in combination with AKT and mTOR is able to significantly decrease the cell viability in both MPNST cell lines analysed, at all tested concentrations (Figure 3A,B). Comparison of triple AKT/mTOR/MEK inhibition with dual AKT/mTOR treatment revealed stronger effects on viability of S462sp cells (*p* < 0.0001) (Figure 3C,D).

### 2.4. Dual Targeting of AKT and mTOR does not Inhibit MPNST Growth In Vivo

**Figure 4:**
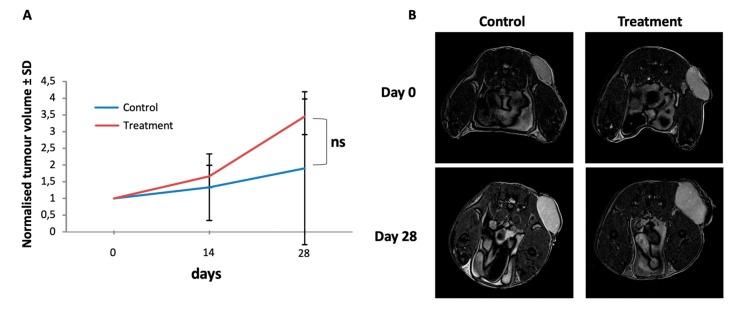
Tumour growth of S462sp cells in a xenotransplantation mouse model is not inhibited by MK-2206 and AZD8055 in vivo. 1 × 10^6^ S462sp cells cultivated in a ratio of 1:1 of neurobasal medium and matrigel were subcutaneously injected into the right flank of SCID mice. After the tumour reached 5 mm in maximum diameter, the mice were randomised into either treatment or control group and treated with either the combination of MK2206 and AZD8055 or vehicle only in the control group. The tumour volume was determined using ultrasound on days 0, 14 and 28 and normalised; the mean values and standard deviation of grouped data from both groups (**A**) are shown. Representative MR images (axial T2w turbo-spin-echo sequence; body array coil at 7T, Bruker ClinScan, Ettlingen, Germany) of MPNST-derived xenograft tumours implanted subcutaneously to the left pelvis of the mice on experimental day 0 and 28 demonstrating significant tumour growth in both the treatment- and the control group, respectively. Since the tumours show a homogenous stroma at both timepoints, pseudo progression due to edema or necrosis can be ruled out (**B**).

To test the effect of the dual AKT/mTOR treatment in vivo, we performed treatment experiments of experimental MPNST in a xenotransplantation mouse model. S462sp cells were subcutaneously implanted into SCID mice and treated for 4 weeks by oral administration of a combination of MK-2206 (80 mg/kg) and AZD8055 (15 mg/kg) once the tumours had reached a diameter of 5 mm. The control group received DMSO vehicle only with the same schedule. The oral administration of drugs was performed by gavage. As measured by ultrasound, the combined treatment of mice with MPNST tumours with MK-2206 and AZD8055 did not have a negative effect on tumour growth kinetics in comparison to the vehicle-treated control group but actually a trend for an increased tumour volume in the treatment group was observed (Figure 4A). Additional measurement of the tumour size by MRI at the beginning and at the end of the treatment period confirmed that the experimental MPNST tumours did not respond to the AKT/mTOR therapy in vivo (Figure 4B). 

### 2.5. Treatment-Resistant MPNST Tumours Have Restored AKT/GSK3b Signaling 

**Figure 5:**
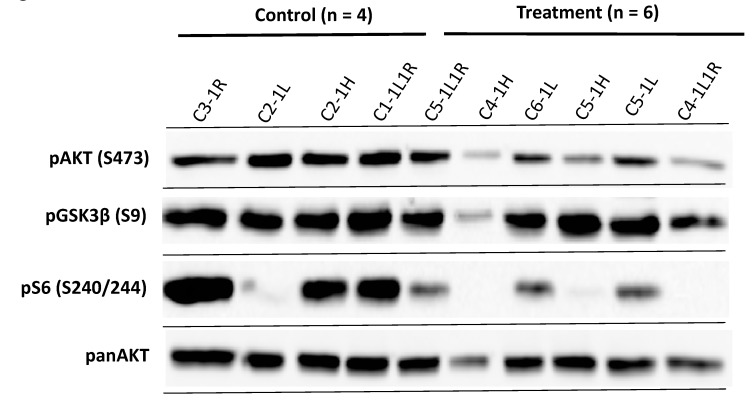
Western blot analysis of MPNST S462sp xenograft tumours confirms the activity of AKT and mTOR inhibitors in vivo. Mice in both groups bearing S462sp xenograft tumours (as shown in Figure 4) were sacrificed after 28 days and tumours carefully excised. The protein lysates were prepared as described previously [25] and probed in a western blot analysis with primary antibodies targeting phospho-AKT (S473), panAKT, phospho-GSK3ß (S9) and phospho-S6 (S240/244).

In order to analyse the possibility that the failure of AKT/mTOR inhibition on xenotransplanted MPNST cells in vivo is due to restoration of AKT and mTOR signaling, we performed western blot analysis of the tumours. Inhibition still occurs in some of the tumours with diminished phospho-S6 levels in 3 out of 6 samples and impaired phospho-AKT levels in 2 out of 6 samples, indicating that the drugs inhibited their respective pathway in the xenotransplants in the mice in vivo (Figure 5).

The results showed a phosphorylation of AKT and its downstream substrate GSK3ß in all the tumour samples from the MK-2206/AZD8055-treated mice. These data strongly suggest that restoration of AKT and mTOR activity may play a role in the resistance of MPNST xenograft tumours to the treatment with AKT and mTOR inhibitors.

## 3. Discussion

Malignant peripheral nerve sheath tumours are the leading cause of death in patients with neurofibromatosis type 1 [10]. There is no efficacious therapy for MPNST patients, and surgical intervention is rarely curative [5]. Therefore, novel experimental therapies have to be tested and validated in preclinical models of NF1-associated neoplasms. MPNST-derived cell lines, which recapitulate the genetic landscape of their corresponding primary tumour, have been demonstrated in the past to be eligible model systems for the evaluation of novel therapeutic agents or combinations of established anticancer drugs in vitro [26,27,28,29] and, when implanted into immunodeficient mice, also in vivo [15,30,31]. MPNST cell lines have also been shown to contain a subpopulation of cells with stem-like properties (cancer stem-like cells, CSLC), thereby representing the intratumoural heterogeneity of MPNST in cell culture [32]. CSLCs have also been described in other high-grade neuro-oncological disorders such as *Glioblastoma multiforme* [33,34]. CSLCs from nervous system tumours are said to be closely related to the expression phenotype of their respective primary tumour and are often held responsible for the failure of treatment due to their relative resistance to radio- and chemotherapy [32,33,35,36]. Furthermore, MPNST cell cultures enriched in CSLCs such as S462sp have a significantly higher tumour take rate when implanted into immunodeficient mice [30,32]. Using S462sp as an in vivo model system, it was recently demonstrated that treatment of mice bearing orthotopic MPNST with oncolytic herpes simplex virus significantly delays tumour growth and prolongs overall survival [30].

The AKT/mTOR pathway is a critical driver of MPNST malignancy [3,37] and targeting of the PI3K/AKT/mTOR pathway is a promising strategy for the treatment of MPNST [16,17,23]. The mTOR-inhibitor rapamycin has so far been the compound of choice to target mTOR, which exists in two complexes, i.e., mTORC1 and mTORC2. Rapamycin has been shown to inhibit mTORC1 function and needs to be administered chronically in vivo to prevent the assembly of the mTORC2 complex, thereby having only limited influence on the activation of AKT [38,39]. The selective dual mTORC1/2 inhibitor AZD8055 has been shown to exert potent anticancer activity both in vitro and in vivo [20,40]. For NF1-associated tumours, Varin et al. have demonstrated that AZD8055 reduces proliferation of PNF and MPNST cells in vitro [17]. Our in vitro results basically confirmed the observations by Varin et al. [17] and extended their current data showing that combinatorial inhibition with the pan-AKT inhibitor MK-2206 and ATP-competitive mTORC1/2 inhibitor has synergistic effects on MPNST S462sp and S1507.2 cell viability. Nevertheless, when using this drug combination in an MPNST xenograft model with S462sp cells in vivo, the initially promising in vitro results could not be confirmed in the in vivo setting. Johannessen et al. [37] could demonstrate a strong reduction of tumour growth while targeting mTORC1 using rapamycin in vivo. Nevertheless, they also observed that rapamycin-mediated tumour growth inhibition was not accomplished via the generally assumed mechanisms and therefore did not induce apoptosis as expected by inhibition of the mTOR pathway [3,37]. 

One striking finding in our in vivo series was the residual activity of the PI3K/AKT/mTOR signalling pathway in the treated tumours. This may implicate a resistance or escape mechanism of tested S462sp MPNST cell line that can be observed in vivo but not in vitro. This reactivation of the AKT and mTOR was also observed by other groups when targeting mTOR in vitro [41]. An interesting observation from our data was a strong phosphorylation of GSK3ß, which could be detected in five out of six samples in the treatment group. GSK3ß is a well-known AKT substrate but also a key molecule in the canonical Wnt pathway in which GSK3ß is crucial for the regulation of ß-Catenin [42,43]. Phosphorylation of GSK3ß at serine residue 9 inactivates GSK3ß and thereby omits phosphorylation and degradation of ß-Catenin [42]. Matching these findings, another group demonstrated a strong pAKT and pGSK3α/ß signal among different human MPNST cell lines [16]. 

Another possible mechanism of resistance could be an overactivation of the RAS/RAF/MEK/MAPK pathway in the resistant tumour cells that has often been shown to be associated with MPNST in NF-1 [4]. In line with this, recent reports showed that targeting the PI3K/AKT/mTOR pathway resulted in a compensatory increase in the RAS/RAF/MEK/MAPK pathway in primary prostate tumour samples [44], indicating strong crosstalk between those pathways as postulated earlier [45]. We therefore conclude that other pathways such as the Wnt signalling pathway or RAS/RAF/MEK/MAPK signalling pathway may be involved in MPNST drug resistance during the treatment with AKT and mTOR inhibitors. 

One putative solution to overcome the drug resistance may be a triple targeting approach against AKT, mTORC1/2 and MEK1/2 by using abovementioned inhibitors. Treatment with MEK1/2 inhibitors showed impaired cell growth in other tumour entities such as breast cancer in the past [46]. In MPNST-induced tumours, Jessen et al. could successfully inhibit tumour growth in a NF1-/- S462-TY human MPNST xenograft model when targeting with allosteric MEK inhibitor PD0325901 [15], underlining the putative usage of an additional MEK1/2 inhibitor in vivo. Indeed, by using the three inhibitors directed against AKT, mTORC1/2 and MEK1/2 we observed synergistic effects in vitro on cell viability of both MPNST cell lines analysed, in comparison to the dual AKT and mTORC1/2 treatment alone. 

Therefore, triple targeting of MPNST with AKT, mTOR and MEK inhibitors may a valuable approach for the treatment of MPNST and should be further analysed in a MPNST xenotransplantation mouse model in vivo.

## 4. Materials and Methods 

### 4.1. Cell Culture

MPNST-derived cell line S1507.2 has been developed and characterised previously [29,47,48]. Cells were propagated in Dulbecco’s modified Eagle’s medium (DMEM) supplemented with 10% fetal calf serum (FCS), 2 mM L-glutamine and 1 mM sodium pyruvate (all Thermo Fisher Scientific, Dreieich, Germany). S462sp were characterised previously [32] and grown as neurospheres in the absence of serum in neurobasal medium (Thermo Fisher Scientific) supplemented with human recombinant epidermal growth factor (EGF, 20 ng/mL, R&D systems, Wiesbaden, Germany), basic fibroblast growth factor (bFGF, 20 ng/mL, Peprotech, Hamburg, Germany), heparin (32 IE/mL, Ratiopharm, Ulm, Germany) and 1% N2 and 1% B27 supplement (Thermo Fisher Scientific) [36].

### 4.2. Proliferation Assay

Cytotoxicity of the tested compounds was measured as described previously [26]. In brief, MPNST cells were seeded in 96-well-plates at 5000 cells per well and treated with the ATP-competitive mTOR inhibitor AZD8055 (AstraZeneca, Cambridge, UK), the allosteric AKT inhibitor MK-2206 (Selleckchem, Munich, Germany), the allosteric MEK1 and MEK2 inhibitor AZD6244 (AstraZeneca, Cambridge, UK), the combination of MK-2206 and AZD8055, or the combination of all three compounds for 72 h. Cytotoxicity was measured by XTT (2,3-bis(2-methoxy-4-nitro-5-sulfophenyl)-5-[(phenylamino) carbonyl]-2H-tetrazolium hydroxide) assay (Roche, Penzberg, Germany) in quadruplicates.

### 4.3. In Vivo Xenograft Model

All animal experiments were approved by the local authority in Hamburg (permission No. 112/11) and performed according to institutional guidelines of animal husbandry and welfare. S462sp cells (1 × 10^6^ cells in 200 µl neurobasal medium:matrigel = 1:1) were subcutaneously injected into the right flank of Fox Chase SCID mice (CB17/lcr-Prkdcscid/lcrlcoCrl, Charles River, Erkrath, Germany, *n* = 18). Animals were randomized into two groups at a tumour maximum diameter of 5 mm, and treatment (*n* = 8) was started by oral administration of AZD8055 (15 mg/kg, 5 days on, two days off) and MK-2206 (80 mg/kg every other day) in a total volume of 50 µl Captisol^®^ for 28 days. Control animals received DMSO vehicle (*n* = 10) only. No general toxicity was observed in the treatment group. Tumour volume was measured by ultrasound every two weeks as described [49], and a final image of the tumours was taken by magnetic resonance imaging (MRI). No mouse developed more than one tumour. Animals were sacrificed at the end of the treatment, and tumours were excised for further analysis. No mice were lost during this study. 

### 4.4. Western Blot

Western blot for phospho-AKT (S473), AKT, phospho-GSK3ß (S9) and phospho-S6-kinase (S240/S244) was performed as described previously [21] using specific primary antibodies (New England Biolabs, Frankfurt a. M., Germany).

### 4.5. Statistical Analysis

Overall statistical analysis was performed using GraphPad Prism 8 (GraphPad Software Inc., San Diego, CA, USA) using one-way ANOVA followed by Dunnett’s multiple comparisons test or Tukey’s multiple comparisons test where applicable. A *p* value < 0.05 was considered as statistically significant. If not indicated otherwise, the mean values and standard deviation are shown.

### 4.6. Determination of Synergistic Effects

Combination indices (CI) for drug combinations were calculated according to the Chou and Talalay method performed as described previously [22] using CompuSyn software (ComboSyn Inc., Paramus, NJ, USA), and CI values were encoded as described previously [24,50] (very strong synergism CI < 0.1; strong synergism CI 0.1–0.3; synergism CI 0.3–0.7; moderate synergism 0.7–0.85; slight synergism CI 0.85–0.90; nearly additive CI 0.9–1.10; CI values > 1.1 were considered as antagonism) [24].
